# Investigating the frequency of tick-borne infections in a case series of 37 youth diagnosed with pediatric bipolar disorder

**DOI:** 10.3389/frcha.2025.1685016

**Published:** 2025-11-06

**Authors:** Rosalie Greenberg

**Affiliations:** 1 Medical Arts Psychotherapy Associates, P.A., Summit, NJ, United States; 2 Overlook Medical Center, Summit, NJ, United States

**Keywords:** pediatric bipolar disorder, tick-borne illness, lyme disease, *Babesia*, bartonella

## Abstract

**Introduction:**

This retrospective chart review examined 37 youth with pediatric bipolar disorder from a private practice in the Lyme-endemic state of New Jersey, expanding on findings from 27 previously reported cases to explore the potential contribution of tick-borne infections to disease etiology.

**Methods:**

Diagnoses were based on DSM-IV-TR and DSM-V criteria using parent and child interviews, questionnaires, and school reports. Initial screening evaluated for possible PANDAS/PANS, with testing for Group A beta-hemolytic streptococcus, *Borrelia burgdorferi*, *Babesia*, *Bartonella*, and *Mycoplasma pneumoniae*. Lyme disease testing included ELISA, Western Blot (IgM/IgG), and immunoblots, interpreted per CDC guidelines. Other pathogens were assessed via IgM/IgG titers, anti-streptolysin O, anti-DNAase B, fluorescent *in situ* hybridization, and blood cultures. A positive diagnosis required both laboratory evidence and clinician confirmation.

**Results:**

*Babesia* was detected in 51% (19/37), *Bartonella* in 49% (18/37), *Mycoplasma pneumoniae* in 38% (14/37), *Borrelia burgdorferi* in 22% (8/37), and Group A Streptococcus in 19% (7/37). Overall, 92% (34/37) had evidence of tick-borne exposure, with 81% (30/37) meeting both laboratory and clinical criteria.

**Discussion:**

More than three-quarters of the cohort demonstrated confirmed tick-borne infections. Overlaps between bipolar disorder and tick-borne illness—such as immune dysregulation, chronic symptomatology, and responsiveness to treatments like minocycline and anti-inflammatory agents—support further exploration of infectious contributors to pediatric bipolar disorder. While limited by its single-practice retrospective design, these findings suggest that tick-borne pathogens may play a role in the pathogenesis of bipolar symptoms in youth, warranting larger, controlled studies.

## Introduction

Recent decades have brought increased recognition of pediatric bipolar disorder (PBD) as a valid clinical diagnosis (1, 2). Concurrently, the rising incidence of tick-borne infections (TBIs) has prompted growing concern regarding their potential neuropsychiatric and neurocognitive sequelae (3, 4). Parallel to these developments, a substantial body of research has underscored the critical role of immune dysregulation and inflammatory processes in the pathogenesis of a broad range of psychiatric conditions, including schizophrenia, major depressive disorder, bipolar disorder, anxiety disorders, obsessive-compulsive disorder (OCD), and attention-deficit/hyperactivity disorder (ADHD) (5–17). Some of the strongest evidence has been in the area of mood disorders, both depression and bipolar disorder. A wide array of infectious agents—including bacterial, viral, fungal, and parasitic pathogens—have been implicated as potential initiators of systemic and neuroinflammatory responses. Previous research has specifically identified associations between certain infectious agents—such as *Toxoplasma gondii,* and *Cytomegalovirus* (CMV)—and the development of bipolar disorder (18). Given the growing evidence linking immune activation to psychiatric symptomatology, elucidating the role of underlying infectious triggers represents an important area for ongoing investigation (19, 20).

Another relevant factor when discussing infections and behavioral/emotional change involves breakdown of the blood brain barrier, normally a gatekeeper and filter protecting the brain from toxic substances carried in the circulation. When compromised, pathogens and toxic substances as well as peripheral immune and inflammatory agents such as cytokines and chemokines normally excluded, can enter the central nervous system. Their entry can subsequently interfere with neuronal function, create neuroinflammation and change neurotransmission (21).

Support for the infection–immune hypothesis in childhood neuropsychiatric illness is seen in the clinical recognition of Pediatric Autoimmune Neuropsychiatric Disorders Associated with Streptococcal infections (PANDAS) (22). It is a syndrome first described by Swedo and colleagues in 1998. The cardinal clinical presentation involves the abrupt onset or dramatic exacerbation of obsessive-compulsive disorder symptoms and/or multiple, complex or unusual tics in children, following infection with Group A beta-hemolytic Streptococcus (GABHS) infection. The course is relapsing and remitting and associated with neuropsychiatric symptoms. Subsequently, the broader diagnostic category of Pediatric Acute-onset Neuropsychiatric Syndrome (PANS) was introduced to describe cases of sudden-onset OCD or severely restricted food intake without requiring a documented preceding streptococcal infection (23). Like PANDAS, PANS can encompass a range of additional neuropsychiatric symptoms, including anxiety, emotional lability, irritability, oppositional behavior, aggression, behavioral/developmental regression, psychotic symptoms, sensory sensitivities, sleep disturbances and urinary disturbances, and can result in the development of arthritic joints and additional autoimmune diseases (24). Studies indicates that PANDAS and PANS are due to an autoimmune encephalopathic process in the basal ganglia (25).

Recent case reports have documented children and adolescents who presented with a variety of psychiatric syndromes that appeared to be resistant to standard treatments, including bipolar disorder, schizophrenia, severe obsessive–compulsive disorder, restrictive eating behaviors, functional neurological symptom disorders, severe anxiety, and cognitive regression (26–28). In these cases, the identification of potential underlying contributors—such as occult infections (e.g., group A streptococcal infections, tick-borne diseases, viral, fungal, and parasitic infections), micronutrient deficiencies, mycotoxin exposure, and gastrointestinal dysbiosis—followed by targeted interventions, was associated with marked clinical improvement.

Against this background, the clinical features of PANDAS/PANS overlap significantly with those observed in pediatric bipolar disorder (PBD), a mental illness characterized by episodic changes in mood, alterations in energy, sleep disruption, cognition, and behavior that impair functioning and can be associated with illness chronicity and significant lifetime morbidity (29).

This paper explores the potential role of tick-borne pathogens including species of *Borrelia, Bartonella, Babesia and Mycoplasma,* in the development or exacerbation of neuropsychiatric symptoms, particularly in the pediatric population.

The first of these illnesses is Lyme disease (LD), the most frequent vector-borne illness in North America and Europe. In the United States, it is caused by the bacteria *Borrelia burgdorferi* and estimated to be responsible for as many as 476,000 new cases each year (30, 31). Approximately 25% of new LD cases occur in youth ages 19 and under, which translates to approximately 119,000 youth/year (32). An Ixodes scapularis tick (the vector) that initiates the infection through its bite may or may not carry *Borrelia* bacteria, but it is also capable of injecting multiple other pathogens into its target. These can include *Babesia, Bartonella, Ehrlichia, Anaplasma, Mycoplasma* species, viruses and other noxious agents. Basically, the tick bite serves as a dirty needle for spread of potentially pathogenic materials.

Historically, discussion of symptoms caused by infectious diseases has focused on the more obvious physical manifestations rather than the effect on mental processes. Recognition that the immune system through mechanisms such as inflammation, immune dysfunction, breakdown of the blood brain barrier, and autoimmunity plays an important role in the development of psychiatric illness and not solely physical disorders, is a rapidly expanding area of scientific study (33). The medical literature refers to the association of microbes and mental illness usually in case reports, and only infrequently focuses on a specific psychiatric illness (20).

This article examines two diagnostic areas, PBD and TBIs, both of which are characterized by unclear symptoms, limited diagnostic methods, and frequent challenges in treatment. According to the American Academy of Pediatrics, around 1%–2% of all U.S. children under 18 have bipolar disorder (34). Other estimates have been higher and up to 5% if one includes the category of bipolar disorder not otherwise specified (35).

The childhood onset of bipolar disorder is associated with significant dysfunction and is a pernicious illness (36–38). It is also a highly heritable disorder. A review of family studies of mood disorders in relatives of individuals with early-onset bipolar disorder indicate these youth have a higher rate of bipolar disorder than relatives of later-onset case, indicating a larger genetic contribution in early-onset forms (39). The risk of bipolar disorder (15%–42%) in first-degree relatives of children with bipolar disorder are consistently larger than the 8.7% estimate of recurrence risk of bipolar disorder in first-degree relatives of adult bipolar disorder cases (40). Genetics is felt to account for 60%–85% of the risk, leaving 15%–40% likely due to other factors. Additional influences include environmental, infectious, immune, and psychosocial factors (41). Multiple adversities early in life can more than double the child's risk of developing bipolar disorder (41). As noted by Post et al. higher genetic loading, increased incidence of childhood abuse and adversity, significant obesity, eating a high inflammatory diet, and limited healthcare access have been factors that contribute to the disorder being a greater problem in the U.S. compared to other countries (42–44).

PBD youth spend more than half (up to 2/3) of their lives struggling in a state of illness, having profound effects on development and the individual's future. It is also important to remember in terms of long-term morbidity that up to 1/3 of children and teens diagnosed with depression in the United States may be experiencing the early onset of PBD (45).

Identifying specific genetic, infectious, and immune vulnerabilities has the potential to identify preventable causes of PBD. Therefore, elucidation of the connection of PBD and TBIs, has the potential to prevent and treat a great deal of illness and suffering.

Rationale for screening pediatric psychiatric patients for a variety of bacterial (including streptococcus bacteria), viral and parasitic pathogens which could be associated with PANDAS or PANS came from the author's observation of the presence of these infections in some very ill and ostensibly treatment resistant psychiatric patients.

The goal of the present study is to further examine the hypothesis that TBIs play a yet unrecognized role in the etiology of some cases of pediatric bipolar disorder (PBD). This retrospective chart review adds an additional 10 pediatric bipolar subjects to 27 youth previously reported on from this author's New Jersey child psychiatry practice [Greenberg, R. (2017). Infections and childhood psychiatric disorders: Tick-borne illness and bipolar disorder in youth. *Bipolar Disorder Open Access, 3*, 113. https://doi.org/10.4172/2472-1077.1000113] (46).

## Methods

In this observational study, a retrospective chart review or 37 youth from a single Northeast U.S. practice in New Jersey, considered by the Center for Disease Control and Prevention as a Lyme and Babesia endemic state, was performed. Rates of exposure to tick-borne pathogens were examined in youth meeting criteria for Bipolar I or II by DSM-IV TR (2000) or DSM-V (2013) (47, 48). The primary difference between the editions is the addition of heightened energy as a criterion for mania in DSM-V, which did not create meaningful divisions between the cohorts. The first chart review of 27 children, conducted prior to the COVID-19 pandemic, used DSM-IV TR, while the second cohort, assessed post-pandemic, used DSM-5V criteria for psychiatric diagnosis. Diagnostic evaluation was consistent across cohorts, with the first assessed in person and the second via a combination of telepsychiatry and in-person visits based on post-COVID practices. The approach utilized in this study was an aggregate data analysis of 37 cases.

All patients underwent a full assessment which included details of the presenting problem, pregnancy, birth and developmental history, school history, social history, first, second and third degree of family history of autoimmune disorders, neurological disorders, cardiovascular disease, medical illnesses, as well as mental disorders including depression, bipolar disorder, anxiety disorders, obsessive compulsive disorders, autism, schizophrenia, ADHD, eating disorders, alcohol abuse, substance abuse, suicide attempts or completed suicide. Inclusion criteria required that patients had received a diagnosis of PBD and had undergone laboratory testing for evidence of exposure to *Streptococcus species, Borrelia, Bartonella, Mycoplasma pneumoniae*, or *Babesia* spp. Exclusion criteria included patients with alternative primary psychiatric diagnoses and those who had not undergone pathogen testing.

Patient blood samples were evaluated for exposure to:

*Borrelia burgdorferi*, the causative agent of LD. Testing included Immunofluorescent Antibody testing (IFA), Enzyme-Linked Immunosorbent Assay (ELISA), Western Blot Immunoglobulins M and G antibody levels, immunoblots and culture techniques. Center for Disease Control and prevention criteria was used for diagnosis.
*Babesia, Bartonella,* and *Mycoplasma pneumoniae*, pathogens that are all potentially carried by ticks. IgM/IgG antibody titers and in some cases fluorescent *in situ* hybridization (FISH) testing were utilized.
*Group A Beta Hemolytic Streptococcal* bacteria. Even though ticks are not known to carry this type of bacteria, the information in this paper was obtained as part of a PANDAS/PANS workup. Streptococcal throat cultures, measurement of anti-streptolysin O titers and anti-strep DNAase B titers were reviewed. Having a PANDAS with comorbid TBI's may be important in illness presentation, course, and treatment.

Laboratories used: LabCorp, Quest Diagnostics, Mayo Medical, IgeneX Laboratory, Advanced Labs, and Galaxy Diagnostics.

Individuals with positive laboratory results were referred for medical evaluation. Clinical confirmation of tick-borne infections (TBIs) was conducted by physicians from diverse specialties, including pediatrics, family medicine, infectious disease, immunology, and rheumatology. All participating clinicians had prior experience with symptom pattern recognition and assessing treatment response in the context of TBIs. Final diagnoses were based on a comprehensive review of the patient's medical history, physical examination findings, and laboratory results.

Noted was the family history of bipolar disorder and depression in the family history of the probands, which included siblings, parents and grandparents, given the issue of heritability.

## Results

Characteristics of the group are seen in Table 1.

**Table 1:** Characteristics of the pediatric bipolar disorder sample (*N* = 37).

Characteristic	Result
Sex	*n* = 10 females (27%); *n* = 27 males (73%)
Mean age (years) at first visit	7.9 (range 2.75 y.o.–16 y.o.)
Age range at PBD diagnosis (years)	3.3–16 y.o.
History of known tick bite	*n* = 3 (8%)
PBD^a^ subtype	PBD I: *n* = 21 (57%); PBD II: *n* = 16 (43%)
+FH^b^ in 1st to 3rd generation relatives (sibs, parents and grandparents)^c^	
Bipolar disorder	*n* = 12
Depression	*n* = 25

a PBD = pediatric bipolar disorder.

b FH = family history.

c Detailed family history was absent in 2 adopted youth in the sample but in one case it was known that both parents had bipolar disorder and a history of substance abuse. The applicable information was included in the above statistics.

**Table 2:** Prevalence of different infections among 37 patients with pediatric bipolar disorder^a^.

Pathogen	Number of positive cases	Percentage positive
*Babesia*	19	51%
*Bartonella* spp.	18	49%
*Mycoplasma pneumoniae*	14	38%
*Borrelia burgdorferi* (LD)	8	22%
*Group A beta-hemolytic Streptococcus*	7	19%

a 14 individuals tested positive for 1 pathogen; 13 were positive for two pathogens; and 7 showed evidence of exposure to 3 pathogens.

The mean age of the 37 subjects was 7.9 years with 27% (10/37) females and 73% (27/37) males. Diagnostically using DSM IV TR and DSM V 57% (21/37) met the criteria for bipolar disorder I and 43% (16/37) were considered bipolar disorder II.

In only three cases of the 37 was there a history of a known tick bite, making this exposure a previously unknown occurrence in at least 31 of the 34 cases (91%) that were positive on testing.

Given that the data was from a private practice and not designed as a clinical study, the timing of the TBI testing relative to the bipolar diagnosis depended upon clinician judgment.

Of note 92% (34/37) of PBD youth were positive on blood testing for TBI exposure. Since this was a case series, there was no standardized protocol for evaluating tick-borne illness (TBI). Over 10 different clinicians conducted assessments based on the recommendation of the author or patient's existing provider or parental choice. Evaluations relied on clinician experience, general medical guidelines, and pattern recognition, reflecting real-world variability in care. Despite this heterogeneity, 88% (30/34) of lab-positive patients were clinically confirmed as having a TBI. Of the four-remaining lab-positive cases two were considered negative by clinician evaluation while the other two families did not wish to seek a consultation. Discordant cases were recorded but not adjudicated.

Several individuals tested positive for more than one pathogen, which is why the cumulative percentages for each pathogen total 179% (Table 2). Specifically, 59% of those with positive results (20/34) had multiple tick-borne infections. All 34 patients with positive testing were referred for clinical evaluation; in two cases, parents declined further assessment. Additionally, two cases were not confirmed as positive after physician review. Overall, clinical evaluation—including history, physical examination, and laboratory results—confirmed TBIs in 81% of the total sample (30/37). Two patients had begun treatment for TBIs prior to receiving a PBD diagnosis. Observing that several newly diagnosed bipolar patients had TBIs led to extending testing to older patients with established PBD, with positive results occurring up to 12 years after the initial psychiatric diagnosis.

## Discussion

In this study greater than 75% of the PBD sample showed evidence of one or more TBIs on testing and clinical exam. At present there is an ongoing controversy about the significance of test results in TBIs, especially LD (49–51).

Although LD is the most studied of the various TBIs, unlike in diabetes mellitus where the accuracy of the testing is very good, standard TBI testing has been considered no better than a coin toss. Emphasis on clinical judgment has been utilized to make the proper diagnosis. Better techniques, especially those focusing on direct detection of the pathogen (*Borrelia burgdorferi*) rather than indirect techniques are sorely needed. Present indirect techniques which are mainly based on demonstrating the presence of antibodies in the blood or other body fluids are subject to a variety of limitations, including using an immune based test to identify a pathogen that is itself immunosuppressive (*Borrelia burgdorferi*, *Bartonella*) (52, 53). Newer techniques such as FISH testing provide direct proof of the presence of the pathogen. In this case series, a diagnosis of TBI was confirmed only when both laboratory testing and clinical assessment were positive. This dual-confirmation approach was implemented to minimize bias that could arise from relying solely on either laboratory results or clinical judgment in isolation. It should be noted in general that not 100% of individuals with a streptococcal infection mount a positive immune response. Also, testing for a streptococcal infection through indirect antibody testing may be negative if there are co-occurring TBIs that have compromised the immune system's ability to make antibodies or if there is baseline immune dysfunction.

What factors are common to both bipolar disorder and TBIs?

Immune dysfunction plays a role in some individuals with bipolar disorder and in those with chronic TBIs e.g., LD/*Bartonella* (14, 52, 53).^,^
Minocycline, a tetracycline antibiotic with anti-inflammatory effects has shown some efficacy in treating both disorders (54, 55).
Both disorders can be chronic and often marked by intermittent exacerbations and remission of the symptoms. This chronicity can make these illnesses harder to treat and is associated with significant morbidity and mortality (3, 56, 57).
Both bipolar disorder and chronic Lyme disease (LD) are associated with depression, suicidal ideation, and increased risk of suicide, with early onset conferring greater long-term morbidity and mortality (58, 59). Notably, a large Danish study found that individuals who contracted LD before age 10 and experienced depression had a higher risk of suicide attempts later in life compared to those without LD or those who contracted it after age 10 (60).
Adults with bipolar disorder who present with rapid cycling or mixed states often demonstrate the greatest resistance to standard treatments (56, 61). Children with PBD frequently exhibit these features and can be challenging to stabilize (62). Could treatment resistant mood symptoms be better explained by the presence of underlying unrecognized infections such as *Borreliosis, Bartonellosis* or *Babesiosis?*
Elevated proinflammatory cytokine levels which can occur in both illnesses have the potential to affect multiple monoamines and other neurotransmitters important in mood disorders (63–65).
Non-steroidal anti-inflammatory agents such as celecoxib and omega 3 fatty acids have been shown to have a possible role in the treatment of bipolar disorder, depression, and LD (66–68),
Both illnesses appear to be multisystem disorders (69–72).
Sleep dysfunction, cognitive dysfunction, symptoms worsening with stress, are all intimately linked to both disorders (73–82).
Increased rates of autoimmune disorders seen in some studies as associated with bipolar disorder, and autoimmune mechanisms (e.g., molecular mimicry) are suspected to play a role in LD pathology (83–88).

The diagnosis of PBD is more common, and bipolar disorder in general has a younger age of onset, in the U.S. compared to many European countries (42). In America, 1/4 of adults with bipolar disorder experience the onset of illness before age 13, and 2/3 before age 19. In many European countries only 1/3 of adults with bipolar disorder have their disorder begin by age 19. These differences suggest that the predominance of *Borrelia burgdorferi* in the U.S., compared with *Borrelia afzelii* and *Borrelia garin*ii in Europe, may contribute to the earlier onset and higher prevalence of PBD among American youth. *Borrelia burgdorferi* is known to trigger a stronger inflammatory response than the European strains. These differences suggest that the predominance of *Borrelia burgdorferi* in the U.S., compared to *Borrelia afzelii* and *Borrelia garinii* in Europe, may in part contribute to the earlier onset and higher prevalence of PBD among American youth (89, 90).

Another factor to consider is that all four of the major tick-borne pathogens, *Borrelia burgdorferi, Babesia, Bartonella and Mycoplasma pneumoniae* have been found to be capable of getting past the blood brain barrier and causing CNS illness (91–94).

Both *Borrelia burgdorferi* and *Babesia* are considered endemic and are reportable infections in New Jersey. Lyme disease, caused by *Borrelia burgdorferi* is transmitted by the blacklegged tick (*Ixodes scapularis*). New Jersey consistently ranks among the states with the highest reported cases, coming in third nationally in 2023 according to the New Jersey State Health Assessment Data (95). Since 2004, the incidence of Lyme disease in New Jersey has more than doubled, rising from 36.1 cases per 100,000 population to 77.8 per 100,000 in 2023 (96, 97).

In comparison to LD, both *babesiosis* and *bartonellosis* remain less extensively studied in humans, and knowledge of their potential long-term effects is limited. The incidence of babesiosis, a parasitic infection that has a preference for invasion of erythrocytes, has increased markedly in New Jersey over the past two decades. In 2004, only 38 cases were reported statewide, whereas 406 cases were documented in 2023 (95, 96).

In contrast, *Bartonella species*, gram-negative intracellular bacteria, are not currently designated as reportable infections in New Jersey, and their transmission by *Ixodes* ticks remains controversial. Despite this, *Bartonella* is often considered a potential co-infection in individuals undergoing diagnostic testing for tick-borne diseases. The Columbia University Lyme and Tick-borne Diseases Research Center note that “the evidence for ticks as vectors of *Bartonella* organisms is circumstantial but fairly strong” (98). Importantly, a growing body of evidence suggests that Bartonella infection may be associated with serious neuropsychiatric and cerebrovascular outcomes, including psychosis, bipolar disorder, schizophrenia, stroke, depression, and anxiety highlighting the need for further research into its clinical and public health impact (26, 99–103).

The generalizability of these findings is limited by several factors. Data were drawn from a single private psychiatric practice located in a Lyme and *Babesia* endemic region, making it unclear whether similar results would be observed in youth with other psychiatric disorders or in non-endemic areas. Retrospective chart reviews in general are limited by incomplete documentation, lack of standardized assessments, temporal ambiguity, and small sample size. Referral bias is also possible given the practice's specialization in pediatric bipolar disorder (PBD), tick-borne infections (TBIs), and PANS/PANDAS. Diagnostic consistency was not standardized, and the use of multiple consultants added variability. In addition, neither psychiatric nor TBI specialists were blinded, as both were aware of clinical diagnoses and laboratory results, introducing potential bias. This non-consecutive case series was further subject to selection bias, since inclusion depended on timing and participants' willingness to participate with different recommendations. Assessments conducted both before and after the COVID-19 pandemic may have influenced results, and unmeasured factors—such as reluctance to provide blood samples—could also have affected interpretation. Given the small sample size and lack of a control group, formal statistical comparisons were not feasible; the data are descriptive and cannot establish causality.

The primary aim was to identify a potential signal—the high frequency of TBI exposure among youth with PBD—that warrants further investigation. Future studies should use prospective designs with psychiatric control groups, standardized diagnostic tools, and systematic data collection to improve reliability and clarify temporal or causal links between pediatric bipolar disorder and tick-borne infections.

Despite these limitations, the study suggests that infections may act as environmental triggers in genetically or immunologically vulnerable individuals. This aligns with prior work implicating infection and immune-related factors in the pathogenesis of bipolar disorder.

Clinically, TBIs should be considered in youth with treatment-resistant psychiatric symptoms, particularly those consistent with PBD. Infections can exacerbate inflammation, compromise blood-brain barrier integrity, and reduce treatment effectiveness, sometimes necessitating targeted interventions. In families with a history of bipolar disorder, infections may act as triggers, revealing underlying genetic vulnerability.

Given the findings of this study, clinicians should consider TBIs in patients whose symptoms are refractory or only partially responsive to standard treatments. Certain infections may provoke inflammatory and immune responses that interfere with conventional therapies, and blood-brain barrier disruption may further contribute to treatment resistance, influencing the choice of interventions such as antibiotics capable of CNS penetration. In cases of PANS or PANDAS presenting with severe, treatment-resistant mood symptoms, assessing the patient's family history for bipolar disorder is important, as infection may unmask genetic susceptibility.

If confirmed, these associations underscore the significance of tick-borne pathogens—including *Borrelia, Bartonella, Babesia*, and *Mycoplasma* species—as potentially modifiable risk factors in pediatric psychiatric illness. Early recognition and treatment could meaningfully reduce the long-term burden of mental illness in vulnerable children.

## Data Availability

The original contributions presented in the study are included in the article/Supplementary Material, further inquiries can be directed to the corresponding author.

## References

[1] Van Meter A Moreira ALR Youngstrom E. Updated meta-analysis of epidemiologic studies of pediatric bipolar disorder. J Clin Psychiatry. (2019) 80(3):18r12180. 10.4088/JCP.18r12180 30946542

[2] Wozniak J O'Connor H Iorini M Ambrose AJH. Pediatric bipolar disorder: challenges in diagnosis and treatment. Paediatr Drugs. (2025) 27(2):125–42. 10.1007/s40272-024-00669-z 39592559 PMC11829910

[3] Bransfield RC. Neuropsychiatric Lyme borreliosis: an overview with a focus on a specialty psychiatrist’s clinical practice. Healthcare (Basel). (2018) 6(3):104. 10.3390/healthcare6030104 30149626 PMC6165408

[4] Brackett M Potts J Meihofer A Indorewala Y Ali A Lutes S Neuropsychiatric manifestations and cognitive decline in patients with long-standing Lyme disease: a scoping review. Cureus (2024) 16(4):e58308. 10.7759/cureus.58308 38752040 PMC11095283

[5] Bechter K. The challenge of assessing mild neuroinflammation in severe mental disorders. Front Psychiatry. (2020) 11:773. 10.3389/fpsyt.2020.00773 32973573 PMC7469926

[6] Leboyer M Berk M Yolken RH Tamouza R Kupfer D Groc L. Immuno-psychiatry: an agenda for clinical practice and innovative research. BMC Med. (2016) 14(1):173. 10.1186/s12916-016-0712-5 27788673 PMC5084344

[7] Raison C. Introduction: the inflammation connection. Psychiatr Times. (2018) 35(5).30820070

[8] Zeng Y Chourpiliadis C Hammar N Seitz C Valdimarsdóttir UA Fang F Inflammatory biomarkers and risk of psychiatric disorders. JAMA Psychiatry. (2024) 81(11):1118–29. 10.1001/jamapsychiatry.2185 39167384 PMC11339698

[9] Ermakov EA Melamud MM Buneva VN Ivanova SA. Immune system abnormalities in schizophrenia: an integrative view and translational perspectives. Front Psychiatry. (2022) 13:880568. 10.3389/fpsyt.2022.880568 35546942 PMC9082498

[10] Khandaker GM Cousins L Deakin J Lennox BR Yolken R Jones PB. Inflammation and immunity in schizophrenia: implications for pathophysiology and treatment. Lancet Psychiatry. (2015) 2(3):258–70. 10.1016/S2215-0366(14)00122-9 26359903 PMC4595998

[11] Jiao W Lin J Deng Y Ji Y Liang C Wei S The immunological perspective of major depressive disorder: unveiling the interactions between central and peripheral immune mechanisms. J Neuroinflammation. (2025) 22(1):10. 10.1186/s12974-024-03312-3 39828676 PMC11743025

[12] Drevets WC Wittenberg GM Bullmore ET Manji HK. Immune targets for therapeutic development in depression: towards precision medicine. Nat Rev Drug Discov. (2022) 21:224–44. 10.1038/s41573-021-00368-1 35039676 PMC8763135

[13] Rosenblat JD McIntyre RS. Bipolar disorder and inflammation. Psychiatr Clin North Am. (2016) 39:125–37. 10.1016/j.psc.2015.09.006 26876323

[14] Oliveira J Oliveira-Maia AJ Tamouza R Brown AS Leboyer M. Infectious and immunogenetic factors in bipolar disorder. Acta Psychiatr Scand. (2017) 136(4):409–23. 10.1111/acps.12791 28832904 PMC7159344

[15] Vogelzangs N Beekman ATF de Jonge P Penninx BWJH. Anxiety disorders and inflammation in a large adult cohort. Transl Psychiatry. (2013) 3:e249. 10.1038/tp.2013.27 23612048 PMC3641413

[16] Marazziti D Mucci F Fontenelle LF. Immune system and obsessive-compulsive disorder. Psychoneuroendocrinology. (2018) 93:39–44. 10.1016/j.psyneuroen.2018.04.017 29689421

[17] Zhou RY Wang JJ Sun JC You Y Ying JN Han XM. ADHD May be a highly inflammation-and immune-associated disease (review). Mol Med Rep. (2017) 16(4):5071–7. 10.3892/mmr.2017.7228 28849096

[18] Frye MA Coombes BJ McElroy SL Jones-Brando L Bond DJ Veldic M Association of *Cytomegalovirus* and *Toxoplasma gondii* antibody titers with bipolar disorder. JAMA Psychiatry. (2019) 76(12):1285–93. 10.1001/jamapsychiatry.2499 31532468 PMC6751798

[19] Stefano GB. Historical insight into infections and disorders associated with neurological and psychiatric sequelae similar to long COVID. Med Sci Monit. (2021) 27:e931447. 10.12659/MSM.931447 33633106 PMC7924007

[20] Bransfield RC Mao C Greenberg R. Microbes and mental illness: past, present, and future. Healthcare (Basel). (2023) 12(1):93. 10.3390/healthcare12010083 38200989 PMC10779437

[21] Grab DJ Perides G Dumler JS Kim KJ Park J Kim YV Borrelia burgdorferi, host-derived proteases, and the blood-brain barrier. Infect Immun. (2005) 73(2):1014–22. 10.1128/IAI.73.2.1014-1022.2005. Erratum in: Infect Immun. 2005 Apr;73(4):2569. 15664945 PMC546937

[22] Swedo SE Leonard HL Garvey M Mittleman B Allen AJ Perlmutter S Pediatric autoimmune neuropsychiatric disorders associated with streptococcal infections: clinical description of the first 50 cases. Am J Psychiatry. (1998) 155:264–71. 10.1176/ajp.155.2.264 9464208

[23] Swedo SE Leckman JF Rose NR. From research subgroup to clinical syndrome: modifying the PANDAS criteria to describe PANS. Pediatr Therapeut. (2012) 2:2. 10.4172/2161-0665.1000113

[24] Ma M Masterson EE Gao J Karpel H Chan A Pooni R Development of autoimmune diseases among children with pediatric acute-onset neuropsychiatric syndrome. JAMA Netw Open. (2024) 7(7):e2421688. 10.1001/jamanetworkopen.2024.21688 39078633 PMC11289697

[25] Leonardi L Perna C Bernabei I Fiore M Ma M Frankovich J Pediatric acute-onset neuropsychiatric syndrome (PANS) and pediatric autoimmune neuropsychiatric disorders associated with streptococcal infections (PANDAS): immunological features underpinning controversial entities. Children (Basel). (2024) 11(9):1043. 10.3390/children11091043 39334578 PMC11430956

[26] Greenberg R. The role of infection and immune responsiveness in a case of treatment-resistant pediatric bipolar disorder. Front Psychiatry. (2023) 14:1074416. 10.3389/fpsyt.2023.1074416 PMC5439003 28588506

[27] Breitschwerdt EB Greenberg R Maggi RG Mozayeni BR Lewis A Bradley JM. Bartonella henselae bloodstream infection in a boy with pediatric acute-onset neuropsychiatric syndrome. J Cent Nerv Syst Dis. (2019) 11:1179573519832014. 10.1177/1179573519832014 30911227 PMC6423671

[28] Monarch ES Foss S. Case report: special recoveries from major mental illness in childhood: a case series. Front Child Adolesc Psychiatry. (2025) 4:1377547. 10.3389/frcha.2025.1377547 40487009 PMC12142067

[29] Greenberg R. Pediatric autoimmune neuropsychiatric disorders associated with streptococcal infections/pediatric acute-onset neuropsychiatric syndromes vs. Pediatric bipolar disorder—a possible connection? Neurol Psychiatry Brain Res. (2014) 20(3):49–54. 10.1016/j.npbr.2014.06.004 ISSN 0941-9500.

[30] Mead P. Epidemiology of Lyme disease. Infect Dis Clin North Am. (2022) 36(3):495–521. 10.1016/j.idc.2022.03.004 36116831

[31] Kugeler KJ Schwartz AM Delorey MJ Mead PS Hinckley AF. Estimating the frequency of lyme disease diagnoses, United States, 2010–2018. Emerg Infect Dis. (2021) 27(2):616–9. 10.3201/eid2702.202731 33496229 PMC7853543

[32] Sood SK. Lyme disease. Pediatr Infect Dis J. (1999) 18(10):913–25. 10530592 10.1097/00006454-199910000-00017

[33] Rosenblat JD McIntyre RS. Bipolar disorder and immune dysfunction: epidemiological findings, proposed pathophysiology and clinical implications. Brain Sci. (2017) 7(11):144. 10.3390/brainsci7110144 29084144 PMC5704151

[34] American Academy of Pediatrics. HealthyChildren.org. (2023). Accessed from Available online at: https://www.healthychildren.org

[35] Merikangas K Cui L Kattan G Carlson G Youngstrom E Angst J. Mania with and without depression in a community sample of U.S. Adolescents. Arch Gen Psychiatry. (2012) 69(9):943–51. 10.1001/archgenpsychiatry.2012.38 22566563 PMC11955849

[36] Post RM Altshuler LL Kupka R McElroy SL Frye MA Grunze H 25 Years of the international bipolar collaborative network (BCN). Int J Bipolar Disord. (2021) 9(1):13. 10.1186/s40345-020-00218-w 33811284 PMC8019011

[37] Post RM Altshuler L Kupka R McElroy S Frye MA Rowe M More pernicious course of bipolar disorder in the United States than in many European countries: implications for policy and treatment. J Affect Disord. (2014) 160:27–33. 10.1016/j.jad.2014.02.006 24709019

[38] Post RM Altshuler LL Kupka R McElroy SL Frye MA Rowe M Age at onset of bipolar disorder related to parental and grandparental illness burden. J Clin Psychiatry. (2016) 77(10):e1309–15. 10.4088/JCP.15m09811 27631141

[39] Faraone SV Glatt SJ Tsuang MT. The genetics of pediatric-onset bipolar disorder. Biol Psychiatry. (2003) 53(11):970–7. 10.1016/s0006-3223(02)01893-0 12788242

[40] Mick E Faraone SV. Family and genetic association studies of bipolar disorder in children. Child Adolesc Psychiatr Clin N Am. (2009) 18(2):441–53, x. 10.1016/j.chc.2008.11.008 19264272

[41] Palmier-Claus JE Berry K Bucci S Mansell W Varese F. Relationship between childhood adversity and bipolar affective disorder: systematic review and meta-analysis. Br J Psychiatry. (2016) 209(6):454–9. 10.1192/bjp.bp.115.179655 27758835

[42] Post RM Altshuler LL Kupka R McElroy SL Frye MA Rowe M More childhood onset bipolar disorder in the United States than Canada or Europe: implications for treatment and prevention. Neurosci Biobehav Rev. (2017) 74(Pt A):204–13. 10.1016/j.neubiorev.2017.01.022 28119069

[43] Post RM Grunze H. The challenges of children with bipolar disorder. Medicina (Kaunas). (2021) 57:601. 10.3390/medicina57060601 34207966 PMC8230664

[44] Post RM Luckenbaugh DA Leverich GS Altshuler LL Frye MA Suppes T Incidence of childhood-onset bipolar illness in the USA and Europe. Br J Psychiatry. (2008) 192(2):150–1. 10.1192/bjp.bp.107.037820 18245035

[45] Geller B Fox LW Clark KA. Rate and predictors of prepubertal bipolarity during follow-up of 6- to 12-year-old depressed children. J Am Acad Child Adolesc Psychiatry. (1994) 33(4):461–8. 10.1097/00004583-199405000-00003 8005898

[46] Greenberg R. Infections and childhood psychiatric disorders: tick-borne illness and bipolar disorder in youth. Bipolar Disorder Open Access. (2017) 3:113. 10.4172/2472-1077.1000113

[47] American Psychiatric Association. Diagnostic and Statistical Manual of Mental Disorders. 4th ed., text rev. Washington, DC: American Psychiatric Association (2000).

[48] American Psychiatric Association. Diagnostic and Statistical Manual of Mental Disorders. 5th ed. Washington, DC: American Psychiatric Association (2013).

[49] Stricker RB Johnson L. Lyme wars: let’s tackle the testing. Br Med J. (2007) 335(7628):1008. 10.1136/bmj.39394.676227.BE PMC2078675 18006976

[50] Stricker RB Johnson L. Lyme disease diagnosis and treatment: lessons from the AIDS epidemic. Minerva Med. (2010) 101(6):419–25. 21196901

[51] Schutzer SE Body BA Boyle J Branson BM Dattwyler RJ Fikrig E Direct diagnostic tests for lyme disease. Clin Infect Dis. (2019) 68(6):1052–7. 10.1093/cid/ciy614 30307486 PMC6399434

[52] Elsner RA Hastey CJ Olsen KJ Baumgarth N. Suppression of long-lived humoral immunity following *Borrelia burgdorferi* infection. PLoS Pathog. (2015) 11(7):e1004976. 10.1371/journal.ppat.1004976 26136236 PMC4489802

[53] Kaufman DL Kogelnik AM Mozayeni RB Cherry NA Breitschwerdt EB. Neurological and immunological dysfunction in two patients with Bartonella *henselae* bacteremia. Clin Case Rep. (2017) 5(6):931–5. 10.1002/ccr3.977 28588842 PMC5458018

[54] Savitz J Preskorn S Teague TK Drevets D Yates W Drevets W. Minocycline and aspirin in the treatment of bipolar depression: a protocol for a proof-of-concept, randomized, double-blind, placebo-controlled, 2 × 2 clinical trial. BMJ Open. (2012) 2:e000643. 10.1136/bmjopen-2011-000643 22357572 PMC3289990

[55] Carris NW Pardo J Montero J Shaeer KM. Minocycline as a substitute for doxycycline in targeted scenarios: a systematic review. Open Forum Infect Dis. (2015) 2(4):ofv178. 10.1093/ofid/ofv178 26719847 PMC4690502

[56] McElroy SL Keck PE Pope HG Hudson JI Faedda GL Swann AC. Clinical and research implications of the diagnosis of dysphoric or mixed mania or hypomania. Am J Psychiatry. (1992) 149:1633–1633. 10.1176/ajp.149.12.1633 1359799

[57] Tsuboi T Suzuki T Azekawa T Adachi N Ueda H Edagawa K Factors associated with non-remission in bipolar disorder: the multicenter treatment survey for bipolar disorder psychiatric outpatient clinics (MUSUBI). Neuropsychiatr Dis Treat. (2020) 16:881–90. 10.2147/NDT.S246136 32280229 PMC7127845

[58] Cirone C Secci I Favole I Ricci F Amianto F Davico C What do we know about the long-term course of early onset bipolar disorder? A review of the current evidence. Brain Sci. (2021) 11(3):341. 10.3390/brainsci11030341 33800274 PMC8001096

[59] Post RM Leveric GS Kupka R Keck PE Jr McElroy SL Altshuler LL Increases in multiple psychiatric disorders in parents and grandparents of patients with bipolar disorder from the USA compared with The Netherlands and Germany. Psychiatr Genet. (2015) 25(5):194–200. 10.1097/YPG.0000000000000093 26146875

[60] Fallon BA Madsen T Erlangsen A Benros ME. Lyme borreliosis and associations with mental disorders and suicidal behavior: a nationwide Danish cohort study. Am J Psychiatry. (2021) 178(10):921–31. 10.1176/appi.ajp.2021.20091347 34315282

[61] Muneer A. Mixed states in bipolar disorder: etiology, pathogenesis and treatment. Chonnam Med J. (2017) 53(1):1–13. 10.4068/cmj.2017.53.1.1 28184334 PMC5299125

[62] Birmaher B. Bipolar disorder in children and adolescents. Child Adolesc Ment Health. (2013) 18(3):140–8. 10.1111/camh.12021 24273457 PMC3835470

[63] Saggu S Pless A Dew E Ware D Jiao K Wang Q. Monoamine signaling and neuroinflammation: mechanistic connections and implications for neuropsychiatric disorders. Front Immunol. (2025) 16:1543730. 10.3389/fimmu.2025.1543730 40356905 PMC12066344

[64] Miller AH Haroon E Raison CL Felger JC. Cytokine targets in the brain: impact on neurotransmitters and neurocircuits. Depress Anxiety. (2013) 30(4):297–306. 10.1002/da.22084 23468190 PMC4141874

[65] Goldstein BI Young LT. Toward clinically applicable biomarkers in bipolar disorder: focus on BDNF, inflammatory markers, and endothelial function. Curr Psychiatry Rep. (2013) 15(12):425. 10.1007/s11920-013-0425-9 24243532 PMC3926699

[66] Arabzadeh S Ameli N Zeinoddini A Rezaei F Farokhnia M Mohammadinejad P Celecoxib adjunctive therapy for acute bipolar mania: a randomized, double-blind, placebo-controlled trial. Bipolar Disord. (2015) 17:606–14. 10.1111/bdi.12324 26291962

[67] Stoll AL Severus WE Freeman MP Rueter S Zboyan HA Diamond E Omega 3 fatty acids in bipolar disorder: a preliminary double-blind, placebo-controlled trial. Arch Gen Psychiatry. (1999) 56:407–12. 10.1001/archpsyc.56.5.407 10232294

[68] Vöckel J Markser A Wege L Wunram HL Sigrist C Koenig J. Pharmacological anti-inflammatory treatment in children and adolescents with depressive symptoms: a systematic-review and meta-analysis. Eur Neuropsychopharmacol. (2024) 78:16–29. 10.1016/j.euroneuro.2023.09.006 37864981

[69] Leboyer M Soreca I Scott J Frye M Henry C Tamouza R Can bipolar disorder be viewed as a multi-system inflammatory disease? J Affect Disord. (2012) 141(1):1–10. 10.1016/j.jad.2011.12.049 22497876 PMC3498820

[70] Zafar K Azuama OC Parveen N. Current and emerging approaches for eliminating Borrelia burgdorferi and alleviating persistent Lyme disease symptoms. Front Microbiol. (2024) 15:1459202. 10.3389/fmicb.2024.1459202 39345262 PMC11427371

[71] Maggi RG Mozayeni BR Pultorak EL Hegarty BC Bradley JM Correa M *Bartonella* spp. bacteremia and rheumatic symptoms in patients from Lyme disease-endemic region. Emerg Infect Dis. (2012) 18(5):783–91. 10.3201/eid1805.111366 22516098 PMC3358077

[72] Bush JC Robveille C Maggi RG Breitschwerdt EB. Neurobartonelloses: emerging from obscurity!. Parasites Vectors. (2024) 17:416. 10.1186/s13071-024-06513-1 39369199 PMC11452993

[73] McIntyre RS. Sleep and inflammation: implications for domain approach and treatment opportunities. Biol Psychiatry. (2016) 2016(80):9–11. 10.1016/j.biopsych.04.018 27312233

[74] Harvey AG Talbot LS Gershon A. Sleep disturbance in bipolar disorder across the lifespan. Clin Psychol (New York). (2009) 16(2):256–77. 10.1111/j.1468-2850.2009.01164.x 22493520 PMC3321357

[75] Weinstein ER Rebman AW Aucott JN Johnson-Greene D Bechtold KT. Sleep quality in well-defined Lyme disease: a clinical cohort study in Maryland. Sleep. (2018) 41(6):zsy035. 10.1093/sleep/zsy035 29452400

[76] Huang Y Zhang Z Lin S Zhou H Xu G. Cognitive impairment mechanism in patients with bipolar disorder. Neuropsychiatr Dis Treat. (2023) 10(19):361–6. 10.2147/NDT.S396424 PMC9926924 36798654

[77] Elias LR Miskowiak KW Vale AMO Köhler CA Kjærstad HL Stubbs B Cognitive impairment in euthymic pediatric bipolar disorder: a systematic review and meta-analysis. J Am Acad Child Adolesc Psychiatry. (2017) 56(4):286–96. 10.1016/j.jaac.01.008 28335872

[78] Tager FA Fallon BA Keilp J Rissenberg M Jones CR Liebowitz MR. A controlled study of cognitive deficits in children with chronic Lyme disease. J Neuropsychiatry Clin Neurosci. (2001) 13(4):500–7. 10.1176/jnp.13.4.500 11748319

[79] Rebman AW Bechtold KT Yang T Mihm EA Soloski MJ Novak CB The clinical, symptom, and quality-of-life characterization of a well-defined group of patients with posttreatment Lyme disease syndrome. Front Med. (2017) 4:224. 10.3389/fmed.2017.00224 PMC5735370 29312942

[80] Breitschwerdt EB Maggi RG Nicholson WL Cherry NA Woods CW. *Bartonella* sp. bacteremia in patients with neurological and neurocognitive dysfunction. J Clin Microbiol. (2008) 46(9):2856–61. 10.1128/JCM.00832-08 18632903 PMC2546763

[81] Bäzner E Meyer TD. Does stress play a significant role in bipolar disorder? A meta-analysis. J Affect Disord. (2017) 208:298–308. 10.1016/j.jad.2016.08.063 27794254

[82] Bransfield RC. The psychoimmunology of lyme/tick-borne diseases and its association with neuropsychiatric symptoms. Open Neurol J. (2012) 6:88–93. 10.2174/1874205X01206010088 23091569 PMC3474947

[83] Chen M Jiang Q Zhang L. The prevalence of bipolar disorder in autoimmune disease: a systematic review and meta-analysis. Ann Palliat Med. (2021) 10(1):350–61. 10.21037/apm-20-2293 33440965

[84] Wang LY Chiang JH Chen SF Shen YC. Systemic autoimmune diseases are associated with an increased risk of bipolar disorder: a nationwide population-based cohort study. J Affect Disord. (2018) 227:31–7. 10.1016/j.jad.2017.10.020 29049933

[85] León-Caballero J Pacchiarotti I Murru A Valentí M Colom F Benach B Bipolar disorder and antibodies against the N-methyl-d-aspartate receptor: a gate to the involvement of autoimmunity in the pathophysiology of bipolar illness. Neurosci Biobehav Rev. (2015) 55:403–12. 10.1016/j.neubiorev.2015.05.012 26014349

[86] Yehudina Y Trypilka S. Lyme borreliosis as a trigger for autoimmune disease. Cureus. (2021) 13(10):e18648. 10.7759/cureus.18648 34786243 PMC8578812

[87] Lochhead RB Strle K Arvikar SL Weis JJ Steere AC. Lyme arthritis: linking infection, inflammation and autoimmunity. Nat Rev Rheumatol. (2021) 17:449–61. 10.1038/s41584-021-00648-5 34226730 PMC9488587

[88] Doskaliuk B Zimba O. *Borrelia burgdorferi* and autoimmune mechanisms: implications for mimicry, misdiagnosis, and mismanagement in lyme disease and autoimmune disorders. Rheumatol Int. (2024) 44:2265–71. 10.1007/s00296-024-05580-x 38578312 PMC11424747

[89] Marques AR Strle F Wormser GP. Comparison of Lyme disease in the United States and Europe. Emerg Infect Dis. (2021) 27(8):2017–24. 10.3201/eid2708.204763 34286689 PMC8314816

[90] Strle K Drouin EE Shen S El Khoury J McHugh G Ruzic-Sabljic E Borrelia burgdorferi stimulates macrophages to secrete higher levels of cytokines and chemokines than *Borrelia afzelii* or *Borrelia garinii*. J Infect Dis. (2009) 200(12):1936–43. 10.1086/648091 19909078 PMC2783242

[91] Kim KS. Mechanisms of microbial traversal of the blood-brain barrier. Nat Rev Microbiol. (2008) 6(8):625–34. 10.1038/nrmicro1952 18604221 PMC5206914

[92] Lum M Syritsyna O Spitzer ED Stone R Montecalvo MA Wormser GP. Neurologic manifestations of tick-borne diseases transmitted by deer ticks (*Ixodes scapularis*) in the USA. Curr Trop Med Rep. (2023) 10:213–21. 10.1007/s40475-023-00302-y

[93] Breitschwerdt EB Maggi RG Robveille C Kingston E. *Bartonella henselae*, *Babesia odocoilei* and *Babesia divergens*-like MO-1 infection in the brain of a child with seizures, mycotoxin exposure and suspected Rasmussen’s encephalitis. J Cent Nerv Syst Dis. (2025) 17:11795735251322456. 10.1177/11795735251322456 40083671 PMC11905044

[94] D'Alonzo R Mencaroni E Di Genova L Laino D Principi N Esposito S. Pathogenesis and treatment of neurologic diseases associated with *Mycoplasma pneumoniae* infection. Front Microbiol. (2018) 9:2751. 10.3389/fmicb.2018.02751 30515139 PMC6255859

[95] New Jersey Department of Health. The New Jersey Department of Health’s State Health Assessment Data (NJSHAD) System (2024). Available online at: https://www-doh.nj.gov/doh-shad/indicator/view/LymeDisease.2024.html (Accessed October 7, 2025).

[96] New Jersey Department of Health. New Jersey Reportable Communicable Disease Report (2004). Available online at: https://capemaycountynj.gov/DocumentCenter/View/1755/Communicable-Disease-Report-2004-PDF?bidId= (Accessed October 7, 2025).

[97] New Jersey Department of Health. New Jersey Reportable Communicable Disease Report (2023). Available online at: https://www.nj.gov/health/cd/documents/reportable_disease/webstatistics_2023.pdf?utm_ (Accessed October 7, 2025).

[98] Columbia University Irving Medical Center Lyme and Tick-Borne Diseases Research Center (2025). Available online at: https://www.columbia-lyme.org/bartonellosis (Accessed October 7, 2025).

[99] Delaney S Robveille C Maggi RG Lashnits E Kingston E Liedig C *Bartonella* species bacteremia in association with adult psychosis. Front Psychiatry. (2024) 15:1388442. 10.3389/fpsyt.2024.1388442/38911703 PMC11190357

[100] Lashnits E Maggi R Jarskog F Bradley J Breitschwerdt E Frohlich F. Schizophrenia and *Bartonella* spp. Infection: a pilot case–control study. Vector Borne Zoonotic Dis. (2021) 21(6):413–21. 10.1089/vbz.2020.2729 33728987 PMC8170724

[101] Balakrishnan N Ericson M Maggi RG Breitschwerdt EB. Vasculitis, cerebral infarction and persistent *Bartonella henselae* infection in a child. Parasit Vectors. (2016) 9(1):254. 10.1186/s13071-016-1547-9 27161220 PMC4862072

[102] Flegr J Preiss M Balátová P. Depressiveness and neuroticism in Bartonella seropositive and seronegative subjects — preregistered case-controls study. Front Psychiatry. (2018) 9:314. 10.3389/fpsyt.2018.00314 30061846 PMC6055045

[103] Schaller JL Burkland GA Langhoff PJ. Do Bartonella infections cause agitation, panic disorder, and treatment-resistant depression? MedGenMed. (2007) 9(3):54. 18092060 PMC2100128

